# Unequal Treatment: Racial and Ethnic Disparities in Alcoholism Treatment Services

**Published:** 2006

**Authors:** Laura Schmidt, Thomas Greenfield, Nina Mulia

**Affiliations:** Laura Schmidt, Ph.D., is an assistant adjunct professor at the Institute for Health Policy Studies and the Department of Anthropology, History and Social Medicine, at the University of California at San Francisco, San Francisco, California. She also is an affiliate senior scientist with the Alcohol Research Group, Berkeley, California. Thomas Greenfield, Ph.D., is a director, and Nina Mulia, Dr.P.H., is an associate scientist, both at the Alcohol Research Group, Public Health Institute, Berkeley, California

**Keywords:** health services research, health care availability and access, alcoholism treatment services research, problematic AOD (alcohol and other drug) use, AODU (AOD use) treatment method, treatment outcomes, treatment barriers, minority group, racial differences, ethnic differences, Black, Hispanic, culturally sensitive prevention approach, health care availability and access

## Abstract

Racial and ethnic disparities in alcoholism treatment may exist with respect to treatment need as well as access to, appropriateness, and quality of care. For any given level of alcohol consumption, ethnic minority populations experience more negative consequences of drinking than Whites and therefore have greater treatment needs. Whether access to treatment is more compromised for minority clients than for Whites is a matter of debate. It is clear, however, that ethnic disparities in the quality and appropriateness of alcohol services are ubiquitous. Despite these disparities, treatment often appears to be as successful for minority patients as for Whites. More in-depth investigations are needed to understand why outcomes often are similar despite disparities in treatment.

Racial and ethnic disparities in medical care are the subject of much debate in the United States. Until recently, research in this area largely examined how differences in insurance coverage and socioeconomic status impact access to care. A recent report by the Institute of Medicine, *Unequal Treatment: Confronting Racial and Ethnic Disparities in Health Care* (Smedley et al. 2002), has fundamentally shifted the focus of this debate by pointing to the deep, racially based inequities that also exist in the quality and appropriateness of care. This report cites a large body of published research revealing that, compared with Whites, minorities tend to receive services of inferior quality, are less likely to receive even routine medical services, and ultimately experience poorer outcomes of care. These disparities persist even after taking into account the differences in insurance coverage, income, and education across racial and ethnic groups.

The debate over health care disparities in general also underscores the need for more information on racial and ethnic differences in the receipt of alcoholism treatment services. Some studies have addressed differences in the need for alcoholism treatment and access to care, but much less research has evaluated the quality, appropriateness, and effectiveness of care. This article reviews the evidence of disparities in each of these areas and points to some new directions for this important area of inquiry. This discussion will focus primarily on data obtained for the two largest minority groups in the United States, Blacks and Hispanics.

## Treatment Need: The Growing Significance of Race

The more researchers have learned about racial and ethnic differences in the types and prevalence of alcohol problems, the more they have come to appreciate the significance of these differences. Since the early 1980s, epidemiological studies have documented pronounced variation in drinking practices and alcohol-related problems that translate into important differences in the need for services, including the following:

- In the three largest population groups, the rates of clinically significant alcohol problems tend to be highest among Hispanic and White men, and lowest among Hispanic women (Grant et al. 2004).
- According to the National Alcohol Survey (NAS), Hispanic men have by far the highest rates of experiencing three or more alcohol problems; moreover, these rates are higher for Black men than for White men. Among women, in contrast, rates of three or more alcohol problems are higher for Whites than for Blacks and Hispanics (Galvan and Caetano 2003).
- Life course studies suggest that symptoms of alcohol dependence remain more stable over time among Hispanic men and that both Hispanic and Black men are more susceptible than White men to developing new problems with dependence (Caetano and Kaskutas 1996).
- The clinical presentation of alcohol problems—including the presence of co-occurring disorders or other problems—also varies by race and ethnicity. For example, both Black and Hispanic men experience higher rates of intimate partner violence and cirrhosis mortality than White men (Caetano 2003).

Studies also have found that, for any given level of alcohol consumption, ethnic minority populations experience more negative health and social consequences of drinking (e.g., alcohol-related legal problems) than Whites (Boyd et al. 2003) and therefore have more complex treatment needs. This higher rate of negative consequences may be related to the stress associated with racial discrimination, to the effects of acculturation, or to ethnic differences in how communities respond to people who exhibit risky drinking behaviors. Moreover, public authorities may be more responsive to the disruptive aspects of drinking by minorities than by Whites (Herd 1994; Jones-Webb et al. 1995). Research has not, however, supported the notion that the disproportionate impact of alcohol-related consequences on minority groups results from greater socioeconomic disadvantages in those groups. Thus, studies have found racial/ethnic differences in alcohol problems to be independent of socioeconomic status (Herd 1994; Buka 2002). This means that ethnicity per se significantly influences alcohol consumption and alcohol-related problems. Still, alcohol abuse among minorities who live in severe economic deprivation can have highly adverse effects on health and mortality in those populations. Ultimately, both ethnicity and socioeconomic status play important roles in the adverse consequences of drinking.

Trend studies further underscore the growing significance of race and ethnicity in research on alcoholism treatment need. National surveys have documented that alcohol consumption and alcohol-related problems among White Americans have declined since the mid-1980s (Caetano and Clark 1998). At the same time, however, the levels of alcohol consumption and alcohol-related problems have remained stable or even increased among Blacks and Hispanics. Particularly striking are the increasing rates of alcohol problems among Hispanic men: the proportion of Hispanic men experiencing three or more symptoms of alcohol dependence rose from 9 percent in 1984 to 16 percent in 1995 (Caetano and Clark 1998). Moreover, Hispanic men as well as Black men and women have longer careers of heavy drinking than Whites, even if they begin drinking later in life (Caetano and Kaskutas 1995).

## Is There a Treatment Gap?

The findings summarized in the previous section support a conclusion that the need for alcoholism treatment services differs greatly across racial and ethnic groups in the United States. This leads to the question whether, taking these different treatment needs into account, all ethnic/racial groups equally utilize care. National surveys suggest that overall, only 9 to 16 percent of Americans with alcohol abuse or dependence obtain treatment for their condition (Green-Hennessy 2002; Woodward et al. 1997).

Whether minority problem drinkers are underserved—and if so, to what extent—is unclear. National censuses of the treatment population suggest that Blacks and Hispanics may be overrepresented, at least in publicly funded treatment programs. Population-based surveys, however, reveal a more mixed picture. After adjusting for differences in treatment need, some studies have found no differences in treatment utilization across ethnic groups, whereas other studies have reported significant differences (Weisner and Schmidt 2001). The uncertainty stems at least in part from difficulties in conducting research on this question, including the following:

- Surveys often lack adequate numbers of minority participants to study rare events, such as alcohol and other drug (AOD) abuse treatment episodes.
- National censuses of the treatment population often overrepresent public-sector programs, which may attract more minority clients than private treatment programs. This may impact the accuracy of patient counts broken down by race.
- Other racial and ethnic differences, such as differences in socioeconomic status and severity of alcohol problems, can confound or mask disparities in treatment use.

Alcoholism treatment can be provided in a variety of settings and using different approaches. Most alcoholism treatment services are delivered in mainstream health and social service settings, such as primary care clinics and mental health programs, rather than in specialized AOD treatment programs. Mutual aid programs such as Alcoholics Anonymous (AA) are another commonly used treatment approach. Members of various ethnic groups differ in the types of care they tend to use. For example, Blacks tend to have lower rates of affiliation with mutual aid programs, such as AA, than Whites or Hispanics (Arroyo et al. 1998). Hispanics also report less participation in AA than Whites, but still appear to reap similar benefits (Tonigan et al. 2002). Because specialty, nonspecialty, and mutual aid programs differ in the kinds of help they offer for alcohol problems, the appropriateness of services received by different ethnic groups may vary.

Racial and ethnic differences in the utilization of alcohol services may result from underlying differences in barriers to care. In an analysis of data from the National Longitudinal Alcohol Epidemiologic Study (NLAES), Grant (1997) identified meaningful differences in the reasons why people fail to obtain needed care. Black respondents in the survey were more likely to report that material concerns—such as not knowing how to find services, lacking means to pay, and being unable to obtain child care while in treatment— kept them from seeking help for an alcohol problem. In contrast, non-Black respondents were more likely to cite psychological and social barriers to care, such as concerns about being stigmatized and beliefs that treatment was not effective. Other evidence suggests that geographic variation in the supply of alcohol services disproportionately reduces minority access to care. An interstate comparison of the supply of alcoholism treatment programs showed that the most underserved areas are found in the southern and southwestern regions, which have disproportionately large minority populations (McAuliffe and Dunn 2004).

Although minority groups may experience more material barriers to care than Whites, they may have characteristics that promote their alcoholism treatment use. For example, members of their families or communities may pressure them to stop drinking and seek help. Studies have found that public reactions to hazardous drinking and the extent of social pressure may be more intense in some minority communities (Herd 1988). Moreover, larger proportions of minorities than Whites are mandated to treatment by the criminal justice system (Polcin 1999). In a revealing analysis, Chasnoff and colleagues (1990) examined the rates of compulsory treatment referral among pregnant women in Florida. Toxicological tests found no racial or ethnic differences in the actual use of AODs among these women. Nevertheless, Black women were 10 times more likely than White women to be reported to the authorities for court intervention and compulsory treatment.

It is commonly assumed that differences in insurance coverage account for ethnic and racial disparities in health care access. Indeed, the two most recent rounds of the NAS have documented significant ethnic differences in insurance coverage among Americans in need of alcoholism treatment. In the 1995 and 2000 surveys combined, 41 percent of Hispanics and 28 percent of Black Americans with a current alcohol dependence or abuse diagnosis were uninsured, compared with 19 percent of Whites and others.^1^ Alcoholism treatment is heavily subsidized by government funds, however, which improves access to care for uninsured people. As a result, researchers rarely find that racial or ethnic differences in insurance coverage contribute significantly to alcoholism treatment disparities (Schmidt and Weisner 2005). In one national analysis of uninsured people with AOD abuse problems, however, uninsured Whites were three times more likely to receive AOD abuse treatment than uninsured Blacks (Wu et al. 2003).

## Are There Differences in the Quality and Appropriateness of Care?

As the IOM report on health care disparities has shown, the medical community is expressing growing concern about racial disparities not only in terms of access to health services but also with respect to the quality and appropriateness of the care received. Few studies in the AOD abuse field have explored these issues directly. Sporadic findings from the health services literature, however, demonstrate that research on treatment disparities should be broadened to critically examine these aspects of the treatment process.

### Quality of Care

Analyses of several factors commonly used to define treatment quality strongly suggest that ethnic disparities in the quality of alcohol services are ubiquitous:

- *Rates of treatment engagement and retention among people with alcohol problems*. To date, few empirical studies have directly examined racial or ethnic disparities in alcoholism treatment quality based on this definition. However, difficulties engaging and retaining minority clients in treatment are often noted in the literature on alcoholism treatment outcomes (Petry 2003).
- *“Waiting time”*—the time a person spends on a waiting list before being admitted to an alcoholism treatment program. In national surveys, Blacks have been disproportionately likely to report that they did not enter treatment because of the lengthy waiting period (Grant 1997).
- *Patient satisfaction*, which can be measured using standardized scales. An analysis of Project MATCH, a multisite clinical trial, found that Blacks and Hispanics reported significantly lower satisfaction with alcoholism treatment than Whites (Tonigan 2003). Despite being less satisfied with their care, however, Blacks in this study experienced superior treatment outcomes, as evidenced by lower drinking rates than Whites at 6 and 12 months following treatment.

### Appropriateness of Care

Evidence also suggests that, in contrast to Whites, minorities may receive care that is less appropriate to their needs. For example, research on outcomes reveals that minority patients are less likely to receive specialty treatment and multiple episodes of care even though they often have different needs (e.g., higher unemployment rates and more legal problems) than Whites (Le Fauve et al. 2003).

The value of culturally specific alcoholism treatment is a topic of vigorous debate: Must treatment regimens specially address patients’ racial and ethnic identities in order to be appropriate? According to treatment program data, about one-third of AOD abuse treatment programs nationwide offer specialty services for Blacks and Hispanics, and less than one-fifth offer specialized services for Native Americans. Many treatment providers assume that culturally sensitive programs are not only more effective but also better able to engage and retain minority clients (Petry 2003). Some findings appear to support this assumption. For example, regional comparisons have indicated that communities offering more services with bilingual and bicultural staff have higher utilization rates among minorities (Rouse et al. 1995).

Culturally specific programs might be more appropriate for several reasons. First, clinical trials increasingly indicate that different racial and ethnic groups may take different pathways to recovery from alcohol problems (Le Fauve et al. 2003). Other findings imply that these groups sometimes vary in their responses to standard treatment approaches. For example, Native American patients in the Project MATCH study experienced better outcomes from motivational enhancement therapy than from cognitive-behavioral therapy or 12-step facilitation (Villanueva et al. 2002). Investigators also may find variations in treatment response within important minority subpopulations, such as those defined by age or co-occurring disorders, but as yet little research has been conducted on these groups (for an exception, see Venner et al. 2003). All these observations suggest that more effective treatment regimens could be developed by taking into account racial and ethnic characteristics.

## Treatment Effectiveness

A few clinical trials and outcome studies have analyzed the effectiveness of treatment for different racial and ethnic groups. Most of these studies have compared Whites with the largest minority groups, primarily Blacks. Although some studies reported poorer treatment outcomes for minority patients, most found no significant differences in treatment effectiveness across groups, based on measures of alcohol consumption following treatment (Brower and Carey 2003). In many studies, minority patients enter treatment with more characteristics that predict lower rates of success (e.g., lower income, less education, more extensive family histories of alcoholism, more co-occurring drug abuse, and poorer physical health) compared with Whites (Le Fauve et al. 2003). Even with poorer odds of success at the beginning of treatment, however, minority patients often appear to be as successful as Whites when followed for a year or more after treatment.

Treatment outcome researchers also have investigated claims that culturally specific treatment programs, as well as therapist–client matching on race, are more effective for minority patients than standard treatment regimens. To date, these studies have provided little support for the idea that specialized treatment protocols produce superior clinical outcomes (Tonigan 2003). However, this issue has not yet been addressed in a randomized controlled trial using a manual-guided, culturally specific treatment regimen, which could provide the strongest evidence for or against the effectiveness of such an approach (Tonigan 2001). Until such a study is available, it appears reasonable for alcoholism treatment programs to focus on providing a range of treatment modalities and culturally sensitive environments. At a minimum, these efforts may be rewarded with higher rates of engagement and retention among minority patients.

Several important limitations in the outcomes studies conducted to date underscore the need to interpret the results of outcome studies with caution.

- First, the finding that ethnic groups do not differ in treatment outcomes could result from unidentified strengths and coping mechanisms that minorities bring to treatment rather than from the effectiveness of the treatment per se.
- Second, study findings may be biased by the fact that minority patients have a higher probability of being excluded from treatment efficacy trials. These patients disproportionately present to treatment programs with characteristics that often are used as exclusion criteria, such as coexisting psychiatric disorders, heavy drug use, and homelessness (Humphreys and Weisner 2000).
- Third, results could be biased by lower rates of treatment engagement and retention among minority patients—for example, if only the most motivated minority patients remain in a study.
- Finally, the patients grouped together under the standard ethnic designations generally are highly heterogeneous with respect to their biological and cultural characteristics. Therefore, a finding that treatment outcomes do not vary across racial/ethnic categories could result from the fact that the racial/ethnic categories used failed to capture the truly meaningful differences across groups.

## Toward More Equal Treatment

The more alcohol researchers investigate the influence of race and ethnicity, the more they have come to appreciate the importance of racial and ethnic disparities in different aspects of treatment. Studies already have documented differences in treatment need and access across ethnic groups in the United States. However, exploration of potential disparities in the quality, appropriateness, and effectiveness of care is only just beginning. One consideration in all such explorations should be that race and ethnicity per se influence the delivery of alcoholism treatment services. One cannot assume that differences in income, education, and insurance coverage among the groups account for all racial and ethnic disparities in alcoholism treatment. Recognizing the complexity of these issues, the National Institute on Alcohol Abuse and Alcoholism (2001) has developed a strategic plan to promote new research on these issues and offers technical guidance on psychometrically validated measures of alcohol problems in minority populations.

The findings discussed in this article underscore the fact that disparities in access to care may not be easily captured in crude national statistics, such as reports of overall rates of treatment use across racial and ethnic groups or data on the proportion of minority patients in treatment facilities. Many factors influence the process of entering treatment for alcohol problems. For example, ethnic groups tend to report differences in average severity of alcohol dependence, clinical presentation of alcohol problems, pathways to care, and the types of barriers to care they experience. Researchers must account for such differences in order to reveal the actual racial and ethnic disparities in treatment utilization. A case in point is the finding that racial and ethnic disparities in access to alcohol services cannot be explained simply by differences in insurance coverage. Of course, the financial barriers to care can be formidable for minority patients, and insurance coverage clearly differs across problem drinkers of varying ethnic origins. Nevertheless, the availability of insurance coverage is only one piece of the puzzle, and racial and ethnic disparities in alcoholism treatment utilization persist even after the vast differences in insurance coverage are taken into account.

Perhaps the most important, and humbling, conclusion to take from the literature is the realization of how inadequately the potential for racial and ethnic differences in quality, appropriateness, and effectiveness of care for alcohol problems has been explored. The potential significance of these concerns is highlighted by existing studies demonstrating that minorities often have lower rates of treatment engagement, retention, and satisfaction with their care. Other findings indicate that even those ethnic groups that experience more severe alcohol problems may be receiving less specialty care than Whites. The reasons for these results, and their implications, are far from clear. These findings could reflect a greater unmet need among minorities for ancillary services that address logistical, economic, and legal concerns; frustration with language barriers; or dissatisfaction with the perceived lack of cultural sensitivity and knowledge on the part of treatment providers. The variety of possible explanations for current research findings should motivate investigators to undertake studies that address deeper questions about racial and ethnic differences in such factors as clinical needs, experiences of the patient–provider encounter, and responses to treatment.

Ultimately, future studies should elucidate the differences in how recovery from alcohol problems unfolds for patients of varying ethnic origins.

## Footnotes

1 p < .001 based on chi-square test of independence; data available from the authors.
